# Prevalence and socio-demographic correlates of cooking skills in UK adults: cross-sectional analysis of data from the UK National Diet and Nutrition Survey

**DOI:** 10.1186/s12966-015-0261-x

**Published:** 2015-08-05

**Authors:** Jean Adams, Louis Goffe, Ashley J. Adamson, Joel Halligan, Nicola O’Brien, Richard Purves, Martine Stead, Deborah Stocken, Martin White

**Affiliations:** Centre for Diet & Activity Research, MRC Epidemiology Unit, University of Cambridge, Box 285 Institute of Metabolic Science, Cambridge Biomedical Campus, Cambridge, CB2 0QQ UK; Institute of Health & Society, Newcastle University, Baddiley-Clark Building, Richardson Road, Newcastle upon Tyne, NE2 4AX UK; Human Nutrition Research Centre, Newcastle University, William Leech Building, Newcastle upon Tyne, NE2 4HH UK; Institute for Social Marketing, Stirling University, Stirling, FK9 4LA UK

**Keywords:** Food handling, Food preparation, Cookery, Cooking skills, Socioeconomic, Inequalities

## Abstract

**Background:**

Poor cooking skills may be a barrier to healthy eating and a contributor to overweight and obesity. Little population-representative data on adult cooking skills has been published. We explored prevalence and socio-demographic correlates of cooking skills among adult respondents to wave 1 of the UK National Diet and Nutrition Survey (2008–9).

**Methods:**

Socio-demographic variables of interest were sex, age group, occupational socio-economic group and whether or not respondents had the main responsibility for food in their households. Cooking skills were assessed as self-reported confidence in using eight cooking techniques, confidence in cooking ten foods, and ability to prepare four types of dish (convenience foods, a complete meal from ready-made ingredients, a main meal from basic ingredients, and cake or biscuits from basic ingredients). Frequency of preparation of main meals was also reported.

**Results:**

Of 509 respondents, almost two-thirds reported cooking a main meal at least five times per week. Around 90 % reported being able to cook convenience foods, a complete meal from ready-made ingredient, and a main dish from basic ingredients without help. Socio-demographic differences in all markers of cooking skills were scattered and inconsistent. Where these were found, women and main food providers were most likely to report confidence with foods, techniques or dishes, and respondents in the youngest age (19–34 years) and lowest socio-economic group least likely.

**Conclusions:**

This is the only exploration of the prevalence and socio-demographic correlates of adult cooking skills using recent and population-representative UK data and adds to the international literature on cooking skills in developed countries. Reported confidence with using most cooking techniques and preparing most foods was high. There were few socio-demographic differences in reported cooking skills. Adult cooking skills interventions are unlikely to have a large population impact, but may have important individual effects if clearly targeted at: men, younger adults, and those in the least affluent social groups.

**Electronic supplementary material:**

The online version of this article (doi:10.1186/s12966-015-0261-x) contains supplementary material, which is available to authorized users.

## Background

Poor cooking skills may be a barrier to healthy eating and a contributor to overweight and obesity, particularly in low income groups [1–3]. People who lack cooking skills may rely on convenience and pre-prepared foods [4–6]. Similarly, easy access to cheap convenience and pre-prepared foods, may decrease motivation to develop cooking skills or cause existing skills and confidence to atrophy [7].

Poorer cooking skills, less frequent preparation of home-cooked food, and more frequent consumption of pre-prepared foods have been associated with poorer dietary quality and overweight and obesity [4, 8–13]. Growing concern about a perceived lack of cooking skills has led to policy interest in adult cooking skills interventions [14, 15].

Many interventions promoting and teaching cooking skills to adults exist at local level in developed countries [15]. Although some interventions report positive effects on diet [16–18], systematic reviews have found few studies reporting high quality evidence [3, 14]. The current state of the evidence makes it difficult to confirm that cooking skills interventions have a consistent, beneficial impact on diet, or body weight. This could be because: few robust evaluations have been conducted [3], interventions are not adequate to achieve such outcomes [15], good cooking skills are more common than has been assumed, or a combination of these, and other, factors.

Home cooking is a complex phenomenon without an agreed definition [19–21]. For example, preparing spaghetti with Bolognese sauce at home could involve: heating up a pre-prepared ‘ready meal’ in a microwave; boiling dried spaghetti, frying minced-beef and adding a stir-in sauce; or making spaghetti and Bolognese sauce from the basic ingredients of flour, eggs, tomatoes, minced-beef, and vegetables. Self-reported cooking skills may also be unrelated to everyday use of such skills–with individuals choosing not to cook at home because another household member takes responsibility for this, they eat elsewhere, or they do not prioritise time for cooking [7].

The most recent population-representative data on adults’ cooking skills in the UK were collected in 1997. This survey found almost 80 % of women, but only 25 % of men, cooked a main meal on most days of the week–although the definition of ‘cooking a main meal’ was not clear. Overall, there were very few techniques or foods that 90 % or more of adults were confident using and there were clear gender differences, favouring women, in confidence across all techniques and foods [22]. Other studies have confirmed that women tend to spend more time cooking and report more developed cooking skills than men [8, 12, 23–28]. Recent data from low-income UK households found that over 90 % of women and children, and over 80 % of men, lived in a household where the ‘main food provider’ (MFP) could prepare a main meal from basic ingredients without help [9]. Indeed recent data from a range of developed countries suggests that most people eat meals prepared at home on most days [8, 11, 13, 22, 23, 29]. UK surveys have found inconsistent trends in cooking skills by age, suggesting that there are not clear cohort effects in self-reported cooking skill, although there may be in how such skills are used [5, 19, 30]. An inverted U-shaped relationship between self-reported cooking skill and age was reported in a Swiss sample peaking at age 50–59 years in women and 40–49 years in men [12]. The reported relationship between markers of socio-economic position and self-reported cooking skills is inconsistent [22, 23, 25, 31, 32].

We are not aware of any recent, population-representative, data on UK adult cooking skills. The aim of this paper is to provide up-to-date information on the prevalence and socio-demographic correlates of cooking skills in UK adults. As food is often purchased and prepared at a household, rather than individual, level, we also explored cooking skills of the MFP (defined below) in respondents’ households.

## Methods

We conducted a cross-sectional analysis of data from wave 1 of the UK National Diet and Nutrition Survey (NDNS) (2008–9).

### Data source

The NDNS is an annual cross-sectional survey assessing the diet, nutrient intake and nutritional status of the general population aged 18 months and upwards living in private households in the UK [33]. Each year, a nationally representative sample is selected using a multi-stage random probability design.

Households across the UK are selected using a multi-stage probability design to take part in the NDNS. In each wave, a random sample of primary sampling units is selected for inclusion. These are small geographical areas that allow more efficient data collection by enabling it to be geographically focused. Within these primary sampling units, private addresses are randomly selected for inclusion. If, on visiting, it is found that more than one household lives at a particular address, one is randomly selected for inclusion. Within participating households, up to one adult and one child are randomly selected to take part as ‘respondents’. Data collection involves a researcher interview covering socio-demographics and shopping, cooking and eating habits; participant completion of a 4-day food diary; and a nurse visit [34].

In each household that includes an NDNS respondent, the person with the main responsibility for shopping and preparing food is identified and labelled the MFP. When these tasks were shared equally between more than one person, either one is identified as the MFP. The MFP can, and often is, also a NDNS respondent. Therefore, within each household the NDNS respondent and the MFP may be the same individual. When they are available at the time of data collection, information is collected from MFPs via a structured interview. This interview includes MFP cooking skills and confidence.

The NDNS aims to collect data from a sample of 1000 respondents per year: 500 adults (aged 19 years and older) and 500 children (aged 1.5 to 18 years). Wave 1 of the NDNS was conducted in February 2008–March 2009 and included a series of interview questions on cooking skills. Data from the NDNS were obtained from the UK Data Archive–an online resource that makes research data available to the UK research community.

### Inclusion criteria

Respondents were included in the analysis if they: took part in wave 1 of the NDNS; were aged 19 years or older at the time of data collection; and did not report that their ability to cook was limited or prevented due to illness. The MFPs in households of included respondents were included if they also provided an in-person interview. Sixteen MFPs did not provide an in-person interview (during which questions on cooking skills were asked) and were excluded from the analyses.

### Variables of interest

Variables of interest fell into two groups: socio-demographic characteristics of respondents and cooking skills of both respondents and MFP.

#### Socio-demographic variables

Socio-demographic variables of interest were sex, age (collapsed into approximately 15 year age bands for analysis), socio-economic position and MFP status.

Socio-economic position was measured using the National-Statistics Socio-economic Classification (NS-SEC) [35]. This is an occupational classification that we collapsed into three groups (higher and managerial, intermediate, and routine and manual occupations) for analysis, with those not currently in employment classified according to their last main occupation. As per normal procedure, those who had never been employed (*n* = 9) or were unclassifiable (*n* = 10) were included in the routine and manual group (the least affluent group).

#### Cooking skills

Cooking skills were assessed in three ways–confidence in using eight cooking techniques, confidence in cooking ten foods, and ability to prepare four types of dish. The same questions were used to assess skills in respondents and MFPs.

Confidence in using eight cooking techniques was established using the question: “Which, if any, of the following cooking techniques do you feel confident about using?: boiling; steaming or poaching; frying; stir frying; grilling; oven-baking or roasting; stewing, braising, or casseroling; and microwaving.” Questions reported here were read verbatim to respondents by researchers.

Confidence in cooking ten foods was determined using the question: “Which, if any, of the following foods do you feel confident about cooking?: red meat, chicken, white fish (cod, haddock, plaice), oily fish (herring mackerel, salmon), pulses (such as split peas and lentils), dry pasta, rice (savoury), potatoes (not chips), fresh green vegetables (cabbage, spinach, broccoli), and root vegetables (carrots, parsnips).”

For both confidence with cooking techniques and cooking specific foods, techniques and foods were listed on a show-card and respondents and MFPs identified those they felt confident with. Those who spontaneously answered that they were confident with all, or none, were coded as such. It was assumed that if a respondent or MFP did not report feeling confident with a technique or food, then they were not confident with that technique or food.

Ability to prepare four different types of dish was determined using the question “Would you be able to make the following foods and dishes from beginning to end?: convenience foods and ready meals (e.g. frozen pizza, pre-packaged curry & rice); a complete meal from ready-made ingredients (e.g. ready-made sauces and pasta to make spaghetti Bolognese); a main dish from basic ingredients (raw potatoes, raw meat, onions etc.), possibly following a recipe (e.g. shepherd’s pie, curry); and a cake or biscuits from basic ingredients (flour, milk, eggs, etc.), possibly following a recipe.” Response options for each type of dish were: “No, not at all”, “Yes, with a lot of help”, “Yes, with a little help”, and “Yes, with no help at all”. As 89 % of respondents answered “Yes, with no help at all” to the first three of types of dish, leading to small frequencies in some cells, we dichotomised responses into “Yes, with no help at all” and other responses.

Respondents and MFPs were also asked how frequently they prepared a main meal for themselves, or themselves and others in their household. No further information was provided on what constitutes preparing a main meal. Seven response options were available: never, only for special occasions, less than once a week, one or two days a week, some days (3–4 a week), most days (5–6 a week), and every day. To maintain comparability with previous data [22], we dichotomised answers into most days (5 days of the week or more) and less often.

### Statistical analysis

Study weights, provided with NDNS data, were used throughout and all analyses were conducted on weighted data. These weights remove any bias imposed by the method of selecting households and individuals to take part; and reduce any non-response bias at the individual (but not question) level [33]. The use of study weights means that all frequencies are presented as percentages (with 95 % confidence intervals) rather than raw frequencies. There was no missing data for individual respondents.

Logistic regression was used to explore simple differences in frequency and confidence of cooking across socio-demographic variables. In these analyses male, the youngest age group, the most affluent social class and not being an MFP were the reference categories against which other categories were compared. All analyses were conducted in Stata v11.

### Ethics

Ethical approval for wave 1 of the NDNS was obtained from Oxfordshire A Research Ethics Committee. We did not require additional ethical approval for this secondary analysis of anonymised data.

## Results

In total, 509 respondents, and 493 MFPs, from 509 households were included in the analysis. Table 1 shows the distribution of socio-demographic variables of interest. There was an even split of respondents by gender (49.0 %, 95 % confidence intervals: 44.2–53.9 male); median age was 46 years (inter-quartile range: 33–62 years); just over one third of respondents (36.1 %, 95 % CI: 31.4–40.9) were in the routine & manual socio-economic group and around one-fifth (20.1 %, 95 % CI: 16.5–24.3) were in the intermediate socio-economic group; and over two-thirds (67.5 %, 95 % CI: 62.5–72.1) were classified as the MFP in their household.

**Table 1:** Frequency and odds of main meal preparation, National Diet & Nutrition Survey, 2008–09, *n* = 509

Variable & level	Distribution, % (95 % confidence intervals)	Respondent prepares main meal for self ± others 5+ days per week		Main food provider prepares main meal for self ± others 5+ days per week	
		% (95 % confidence intervals)	Odds ratio (95 % confidence intervals)	% (95 % confidence intervals)	Odds ratio (95 % confidence intervals)
All	100	63.1 (58.1–67.8)	--	83.9 (80.0–87.2)	--
Gender					
Men	49.0 (44.2–53.9)	43.5 (36.5–50.8)	Reference	84.5 (79.0–88.8)	Reference
Women	51.0 (46.1–55.8)	81.9 (76.0–86.6)	5.85 (3.69–9.28)	83.4 (77.4–88.0)	0.92 (0.54–1.56)
Age (years)					
19–34	28.0 (23.6–32.8)	53.3 (43.1–63.2)	Reference	76.9 (67.1–84.5)	Reference
35–49	27.6 (23.5–32.0)	67.8 (59.0–75.4)	1.85 (1.06–3.22)	85.6 (79.1–90.3)	1.78 (0.91–3.48)
50–64	24.4 (20.5–28.6)	66.3 (56.4–75.0)	1.73 (0.96–3.10)	87.8 (81.0–92.3)	2.15 (1.05–4.42)
> 64	20.1 (16.5–24.3)	66.3 (55.5–75.6)	1.73 (0.94–3.18)	86.7 (77.2–92.7)	1.96 (0.86–4.47)
NS-SEC					
Managerial & prof.	41.7 (37.0–46.7)	60.2 (52.3–67.5)	Reference	84.0 (77.2–89.1)	Reference
Intermediate	22.2 (18.3–26.8)	72.7 (61.3–81.7)	1.76 (0.96–3.24)	88.4 (79.7–93.7)	1.46 (0.65–3.24)
Routine & manual	36.1 (31.4–40.9)	60.5 (52.2–68.2)	1.01 (0.64–1.61)	81.7 (75.0–86.9)	0.85 (0.47–1.53)
Respondent is main food provider					
No	32.6 (27.9–37.5)	16.9 (11.0–25.1)	Reference	--	--
Yes	67.5 (62.5–72.1)	85.3 (80.8–88.9)	28.48 (15.77–51.44)	--	--

*NS*-*SEC* National Statistics socio-economic classification, -- not applicable

Table 1 also shows the proportion of respondents who reported preparing a main meal at least five times per week, and the proportion who lived in a household where the MFP did this. Almost two thirds of respondents (63.1 %, 95 % CI: 58.1–67.8) said they prepared a main meal on most days of the week, whilst more than four-fifths (83.9 %, 95 % CI: 80.0–87.2) lived in a household where the MFP said they did so. Women, those aged 35–49 years, and respondents who were MFPs were more likely to report cooking a main meal on most days. Individuals aged 50–64 years were more likely to live in a household where the MFP cooked on most days of the week than those aged 19–34 years.

The percentages of respondents reporting confidence in using the eight cooking techniques are shown in Table 2. Three-quarters, or more, of respondents reported confidence with using each of the techniques, except stir-frying (where just less than three-quarters–74.4 %, 95 % CI: 70.0–78.4–reported confidence). At least 90 % of respondents reported confidence with boiling (93.1 %, 95 % CI: 90.0–95.2), grilling (90.6 %, 95 % CI: 87.3–93.1), and oven-baking or roasting (90.0 %, 95 % CI: 86.4–92.7).

**Table 2:** Confidence in using eight cooking techniques, National Diet & Nutrition Survey, 2008–09, *n* = 509

Variable & level	Boiling, % (95 % CI)	Steaming, poaching, % (95 % CI)	Frying, % (95 % CI)	Stir frying, % (95 % CI)	Grilling, % (95 % CI)	Oven-baking, roasting, % (95 % CI)	Stewing, braising, casseroling, % (95 % CI)	Microwaving, % (95 % CI)
All respondents	93.1 (90.0–95.2)	75.0 (70.4–79.0)	88.2 (84.8–90.9)	74.4 (70.0–78.4)	90.6 (87.3–93.1)	90.0 (86.4–92.7)	76.0 (71.4–80.1)	83.1 (79.3–86.4)
Gender								
Men	89.7 (84.2–93.4)	67.3 (60.0–73.9)	90.0 (84.2–93.2)	69.7 (62.6–75.9)	87.7 (82.1–91.8)	81.9 (75.3–87.0)	81.9 (75.8–86.8)	81.9 (75.8–86.8)
Women	96.3 (92.6–98.2)	82.3 (76.9–86.7)	86.8 (82.1–90.4)	79.0 (73.4–83.7)	93.4 (89.4–96.0)	97.7 (95.1–98.9)	84.2 (79.2–88.2)	84.2 (79.2–88.2)
Age (years)								
19–34	92.2 (85.7–95.9)	59.6 (49.3–69.0)	89.0 (82.1–93.4)	72.5 (63.1–80.3)	87.1 (79.1–92.3)	88.8 (80.5–93.8)	59.8 (49.6–69.3)	89.4 (82.0–94.0)
35–49	94.5 (99.1–97.5)	86.5 (79.0–91.6)	88.3 (81.5–92.8)	83.3 (75.5–88.9)	94.8 (88.9–97.6)	96.7 (92.1–98.7)	82.0 (73.9–88.0)	81.3 (73.8–87.0)
50–64	93.0 (84.2–97.0)	78.2 (69.2–85.2)	86.6 (78.5–91.9)	74.5 (65.2–82.0)	92.5 (84.8–96.5)	88.7 (79.8–94.0)	84.1 (75.5–90.1)	82.2 (73.7–88.5)
> 64	92.4 (84.1–96.5)	76.6 (66.7–84.3)	88.7 (80.1–93.9)	64.8 (54.1–74.2)	87.6 (78.9–93.0)	83.8 (74.0–90.4)	80.6 (70.7–87.7)	77.8 (68.0–85.3)
NS-SEC								
Managerial & prof.	93.3 (88.3–96.3)	78.6 (71.4–84.3)	90.4 (85.3–93.8)	81.0 (74.4–86.3)	93.8 (89.2–96.6)	92.0 (86.3–95.5)	76.4 (69.0–82.4)	86.9 (81.3–90.9)
Intermediate	96.0 (88.3–98.7)	86.1 (76.2–92.3)	91.5 (84.1–95.6)	82.1 (72.9–88.7)	94.6 (87.7–97.7)	94.9 (86.8–98.1)	89.9 (80.5–95.1)	84.6 (76.1–90.4)
Routine & manual	91.1 (85.0–94.9)	64.9 (57.1–72.0)	84.0 (77.4–88.9)	63.1 (55.3–70.3)	85.1 (78.2–90.0)	85.0 (78.1–90.0)	67.9 (59.9–74.9)	78.3 (71.1–84.2)
Respondent is main food provider								
No	85.7 (77.9–91.1)	63.2 (53.7–71.9)	89.7 (82.4–94.1)	69.8 (70.6–77.6)	88.2 (80.3–93.2)	79.2 (70.4–85.9)	58.7 (49.1–67.6)	87.5 (79.9–92.5)
Yes	96.6 (93.7–98.2)	80.6 (75.8–84.6)	87.4 (83.4–90.6)	76.7 (71.6–81.1)	91.8 (88.3–94.3)	95.1 (92.1–97.0)	84.4 (79.9–88.1)	81.0 (76.3–84.9)

*CI* confidence intervals, *NS*-*SEC* National Statistics socio-economic classification

Odds of reporting confidence with cooking techniques by socio-demographic variables are shown in Table 3. There were some, scattered, differences in confidence with techniques by socio-demographic variables. Where these were present, women, respondents who were older than 19–34 years, and respondents who were MFPs tended to be most likely to report confidence with cooking techniques. No socio-demographic differences were seen in confidence with frying, or microwaving.

**Table 3:** Odds of having confidence using eight cooking techniques, National Diet & Nutrition Survey, 2008–09, *n* = 509

Variable & level	Boiling, odds ratio (95 % CI)	Steaming, poaching, odds ratio (95 % CI)	Frying, odds ratio (95 % CI)	Stir frying, odds ratio (95 % CI)	Grilling, odds ratio (95 % CI)	Oven-baking, roasting, odds ratio (95 % CI)	Stewing, braising, casseroling, odds ratio (95 % CI)	Microwaving, odds ratio (95 % CI)
Gender								
Men	Reference	Reference	Reference	Reference	Reference	Reference	Reference	Reference
Women	2.97 (1.24–7.08)	2.26 (1.43–2.60)	0.77 (0.42–1.39)	1.64 (1.05–2.55)	1.99 (1.00–3.96)	9.46 (3.94–22.71)	2.61 (1.61–4.25)	1.18 (0.71–1.94)
Age (years)								
19–34	Reference	Reference	Reference	Reference	Reference	Reference	Reference	Reference
35–49	1.43 (0.49–4.20)	4.34 (2.22–8.51)	0.94 (0.43–2.04)	1.89 (0.99–3.61)	2.69 (0.99–7.33)	3.72 (1.20–11.48)	3.06 (1.63–5.74)	0.51 (0.24–1.10)
50–64	1.11 (0.36–3.45)	2.44 (1.31–4.57)	0.80 (0.36–1.78)	1.11 (0.60–2.06)	1.83 (0.68–4.89)	0.99 (0.38–2.56)	3.56 (1.80–7.04)	0.55 (0.25–1.22)
> 64	1.02 (0.35–3.00)	2.22 (1.17–4.22)	0.98 (0.41–2.35)	0.70 (0.37–1.30)	1.05 (0.44–2.47)	0.65 (0.27–1.58)	2.79 (1.41–5.53)	0.42 (0.19–0.92)
NS-SEC								
Managerial & prof.	Reference	Reference	Reference	Reference	Reference	Reference	Reference	Reference
Intermediate	1.74 (0.46–6.54)	1.69 (0.79–3.62)	1.15 (0.49–2.71)	1.07 (0.56–2.08)	1.15 (0.39–3.38)	1.62 (0.49–5.39)	2.76 (1.18–6.48)	0.83 (0.42–1.65)
Routine & manual	0.73 (0.30–1.77)	0.48 (0.29–0.80)	0.54 (0.28–1.04)	0.39 (0.24–0.66)	0.37 (0.17–0.82)	0.49 (0.23–1.06)	0.67 (0.40–1.12)	0.57 (0.32–1.02)
Respondent is main food provider								
No	Reference	Reference	Reference	Reference	Reference	Reference	Reference	Reference
Yes	4.73 (2.06–10.89)	2.42 (1.49–3.93)	0.80 (0.40–1.61)	1.42 (0.88–2.31)	1.50 (0.72–3.09)	5.11 (2.53–10.32)	3.81 (2.33–6.26)	0.61 (0.32–1.14)

*CI* confidence intervals, *NS*-*SEC* National Statistics socio-economic classification

Table 4 shows confidence with cooking 10 different foods. More than three-quarters of respondents reported confidence cooking each food, except oily fish (69.9 %, 95 % CI: 65.3–74.1) and pulses (60.4 %, 95 % CI: 55.5–65.1). More than 90 % of respondents reported confidence with cooking chicken (91.3 %, 95 % CI: 88.1–93.8), potatoes (94.3 %, 95 % CI: 91.2–95.1), and fresh green vegetables (92.7 %, 95 % CI: 89.4–95.1).

**Table 4:** Confidence in cooking 10 foods, National Diet & Nutrition Survey, 2008–09, *n* = 509

Variable & level	Red meat, % (95 % CI)	Chicken, % (95 % CI)	White fish, % (95 % CI)	Oily fish, % (95 % CI)	Pulses, % (95 % CI)	Dry pasta, % (95 % CI)	Rice (savoury), % (95 % CI)	Potatoes (not chips), % (95 % CI)	Fresh green veg, % (95 % CI)	Root veg, % (95 % CI)
All	87.7 (84.2–90.5)	91.3 (88.1–93.8)	79.7 (75.4–83.5)	69.9 (65.2–74.1)	60.4 (55.5–65.1)	84.9 (80.9–88.1)	87.8 (84.1–90.7)	94.3 (91.2–96.4)	92.7 (89.4–95.1)	89.6 (86.1–92.3)
Gender										
Men	87.8 (82.3–91.7)	88.4 (82.7–92.3)	74.9 (67.8–80.9)	63.8 (56.5–70.5)	55.1 (47.7–62.2)	78.8 (72.3–84.2)	83.2 (76.9–88.1)	92.4 (87.0–95.7)	87.9 (81.9–92.1)	85.5 (79.5–90.0)
Women	87.6 (82.6–91.3)	94.2 (90.3–96.6)	84.3 (79.1–88.4)	75.7 (70.0–80.7)	65.4 (58.9–71.4)	90.7 (86.0–93.9)	92.1 (87.9–95.0)	96.2 (92.1–98.2)	97.3 (93.7–98.9)	93.5 (89.2–96.1)
Age (years)										
19–34	82.0 (73.8–88.0)	88.9 (82.0–93.4)	70.5 (60.7–78.7)	56.5 (46.4–65.6)	46.4 (36.6–56.4)	92.3 (85.7–96.0)	91.7 (84.8–95.6)	93.4 (85.8–97.1)	90.4 (82.5–95.0)	85.7 (77.7–91.2)
35–49	91.0 (84.1–95.1)	93.6 (87.2–96.9)	78.7 (70.3–85.3)	78.0 (69.9–84.4)	67.1 (58.4–74.8)	92.0 (84.6–96.0)	93.4 (86.0–97.0)	94.9 (87.6–98.0)	93.1 (85.7–96.8)	92.7 (86.0–96.3)
50–64	91.1 (83.1–95.5)	92.1 (83.3–96.4)	86.2 (77.5–91.9)	74.4 (65.2–81.9)	65.2 (55.6–73.7)	81.9 (72.6–88.5)	84.9 (75.7–91.0)	94.6 (86.4–98.0)	93.1 (84.4–97.1)	90.3 (81.6–95.1)
> 64	87.0 (78.4–92.5)	90.6 (82.1–95.3)	86.0 (76.8–92.0)	71.9 (61.3–80.5)	64.7 (53.9–74.1)	68.3 (57.7–77.4)	78.2 (68.2–85.7)	94.6 (87.3–97.8)	94.9 (87.9–98.0)	90.0 (80.9–95.0)
NS-SEC										
Managerial	88.0 (81.9–92.2)	91.8 (86.3–95.2)	82.4 (75.8–87.5)	76.4 (69.4–82.2)	69.2 (61.7–75.9)	90.2 (84.5–94.0)	91.0 (84.9–94.8)	95.1 (90.3–97.5)	92.9 (87.4–96.1)	90.5 (84.7–94.3)
Intermediate	93.9 (87.3–97.1)	96.0 (88.6–98.7)	85.4 (75.5–91.7)	79.6 (69.4–87.0)	69.3 (58.2–78.5)	85.2 (75.6–91.5)	93.2 (86.5–96.7)	97.5 (84.5–99.6)	99.6 (96.9–99.9)	97.6 (90.9–99.4)
Routine	84.0 (77.4–88.9)	88.2 (82.0–92.5)	73.7 (66.0–80.2)	57.6 (49.8–65.1)	46.0 (38.5–53.7)	79.0 (71.8–84.8)	81.3 (74.2–86.8)	91.8 (85.6–95.5)	88.7 (81.8–93.2)	84.1 (77.2–89.2)
Respondent is main food provider										
No	81.7 (73.5–87.8)	84.3 (76.2–90.0)	67.4 (58.0–75.6)	56.2 (46.8–65.3)	49.5 (40.2–58.8)	81.6 (73.3–87.7)	84.2 (76.0–89.9)	90.0 (82.6–94.4)	83.9 (75.6–89.7)	81.1 (72.7–87.3)
Yes	90.6 (86.9–93.3)	94.7 (91.7–96.7)	85.7 (81.3–89.2)	76.4 (71.5–80.7)	65.6 (59.9–70.8)	86.5 (81.9–90.0)	89.5 (85.4–92.6)	96.5 (92.9–98.3)	97.0 (93.7–98.6)	93.7 (90.2–96.0)

*CI* confidence intervals, *NS*-*SEC* National Statistics socio-economic classification

Odds ratios of confidence with different foods are shown in Table 5. As before, women, those who were older than 34 years, and those who were the MFP tended to be most likely to report confidence. Those in the lowest socio-economic group tended to be least likely to report confidence with cooking specific foods.

**Table 5:** Odds of having confidence in cooking 10 foods, National Diet & Nutrition Survey, 2008–09, *n* = 509

Variable & level	Red meat, odds ratio (95 % CI)	Chicken, odds ratio (95 % CI)	White fish, odds ratio (95 % CI)	Oily fish, odds ratio (95 % CI)	Pulses, odds ratio (95 % CI)	Dry pasta, odds ratio (95 % CI)	Rice (savoury), odds ratio (95 % CI)	Potatoes (not chips), odds ratio (95 % CI)	Fresh green veg, odds ratio (95 % CI)	Root veg, odds ratio (95 % CI)
Gender										
Men	Reference	Reference	Reference	Reference	Reference	Reference	Reference	Reference	Reference	Reference
Women	0.99 (0.55–1.78)	2.12 (1.03–4.36)	1.80 (1.10–2.94)	1.77 (1.16–2.70)	1.54 (1.03–2.32)	2.62 (1.46–4.68)	2.36 (1.27–4.41)	2.06 (0.78–5.46)	4.99 (1.80–13.81)	2.43 (1.21–4.87)
Age (years)										
19–34	Reference	Reference	Reference	Reference	Reference	Reference	Reference	Reference	Reference	Reference
35–49	2.23 (1.00–5.00)	1.83 (0.71–4.76)	1.55 (0.83–2.90)	2.73 (1.52–4.90)	2.36 (1.36–4.09)	0.96 (0.35–2.65)	1.28 (0.44–3.75)	1.33 (0.36–4.82)	1.44 (0.50–4.20)	2.11 (0.86–5.20)
50–64	2.25 (0.94–5.41)	1.44 (0.52–4.00)	2.63 (1.25–5.50)	2.24 (1.23–4.07)	2.17 (1.23–3.83)	0.38 (0.16–0.91)	0.51 (0.21–1.25)	1.24 (0.33–4.65)	1.44 (0.46–4.53)	1.55 (0.62–3.88)
> 64	1.47 (0.68–3.21)	1.20 (0.47–3.07)	2.58 (1.21–5.52)	1.97 (1.06–3.69)	2.12 (1.16–3.87)	0.18 (0.08–0.41)	0.33 (0.14–0.77)	1.24 (0.35–4.37)	1.98 (0.61–6.40)	1.50 (0.59–3.79)
NS-SEC										
Managerial	Reference	Reference	Reference	Reference	Reference	Reference	Reference	Reference	Reference	Reference
Intermediate	2.09 (0.82–5.31)	2.17 (0.61–7.73)	1.25 (0.59–2.65)	1.20 (0.63–2.30)	1.00 (0.56–1.80)	0.63 (0.28–1.41)	1.35 (0.52–3.52)	2.04 (0.25–16.82)	17.51 (2.9–140.23)	4.31 (0.95–19.57)
Routine	0.72 (0.38–1.40)	0.65 (0.30–1.41)	0.60 (0.34–1.05)	0.43 (0.26–0.70)	0.37 (0.23–0.59)	0.37 (0.18–0.71)	0.40 (0.19–0.83)	0.54 (0.20–1.43)	0.61 (0.26–1.47)	0.56 (0.27–1.14)
Respondent is main food provider										
No	Reference	Reference	Reference	Reference	Reference	Reference	Reference	Reference	Reference	Reference
Yes	2.15 (1.18–3.93)	3.36 (1.66–6.82)	2.89 (1.72–4.83)	2.52 (1.60–3.99)	1.94 (1.24–3.04)	1.44 (0.80–2.60)	1.61 (0.85–3.06)	3.04 (1.16–7.99)	6.19 (2.45–15.60)	3.48 (1.77–6.86)

*CI* confidence intervals, *NS*-*SEC* National Statistics socio-economic classification

The reported ability of respondents to prepare four types of dishes without help, and odds of these by socio-demographic group, is shown in Table 6. More than 90 % of respondents reported being able to prepare ready meals (97.6 %, 95 % CI: 95.3–98.7) and a meal from ready-made ingredients (93.1 %, 95 % CI: 90.1–95.3) without help, with 89.2 % (95 % CI: 85.5–92.1) reporting being able to do the same for a main dish from basic ingredients. Just over two thirds of respondents (69.0 %, 95 % CI: 64.2–73.4) said they could bake a cake or biscuits without help. There were few statistically significant differences in ability to prepare these dishes by socio-demographic groups. Where these were found, women and those who were MFPs were most likely to be able to prepare dishes without help.

**Table 6:** Ability, and odds of having ability, to prepare four dish types without help, National Diet & Nutrition Survey, 2008–09, *n* = 509

Variable & level	Convenience foods & ready meals		Complete meal from ready-made ingredients		Main dish from basic ingredients		Cake or biscuits from basic ingredients	
	% (95 % CI)	Odds ratio (95 % CI)	% (95 % CI)	Odds ratio (95 % CI)	% (95 % CI)	Odds ratio (95 % CI)	% (95 % CI)	Odds ratio (95 % CI)
All respondents	97.6 (95.3–98.7)	--	93.1 (90.1–95.3)	--	89.2 (85.5–92.1)	--	69.0 (64.2–73.4)	--
Gender								
Men	95.7 (91.3–97.9)	Reference	89.2 (83.6–93.0)	Reference	82.8 (76.1–87.9)	Reference	47.6 (40.0–54.9)	Reference
Women	99.4 (97.1–99.9)	6.91 (1.28–37.33)	97.0 (94.0–98.5)	3.90 (1.65–9.17)	95.5 (92.1–97.4)	1.10 (0.40–3.03)	89.5 (85.1–92.7)	9.44 (5.77–15.45)
Age (years)								
19–34	98.9 (95.6–99.7)	Reference	95.6 (89.8–98.2)	Reference	86.0 (77.5–91.6)	Reference	64.8 (54.7–73.8)	Reference
35–49	99.2 (94.5–99.9)	1.44 (0.13–16.23)	97.4 (93.0–99.1)	1.71 (0.43–6.80)	91.6 (84.1–95.8)	1.79 (0.71–4.51)	79.1 (70.9–85.4)	2.05 (1.11–3.77)
50–64	94.5 (85.9–98.0)	0.20 (0.03–1.12)	90.4 (81.6–95.3)	0.43 (0.13–1.41)	89.9 (81.0–95.0)	1.46 (0.57–3.73)	67.8 (57.8–76.4)	1.14 (0.63–2.09)
> 64	97.2 (91.1–99.2)	0.39 (0.06–2.53)	87.1 (78.1–92.8)	0.31 (0.10–0.94)	89.5 (80.8–94.6)	1.39 (0.56–3.49)	62.4 (51.5–72.1)	0.87 (0.47–1.61)
NS-SEC								
Managerial & prof.	97.9 (94.5–99.2)	Reference	94.2 (89.4–96.9)	Reference	92.3 (87.0–95.6)	Reference	73.5 (66.1–79.7)	Reference
Intermediate	98.1 (92.6–99.5)	1.12 (0.19–6.43)	94.2 (85.1–97.8)	0.98 (0.29–3.37)	88.5 (77.9–94.4)	0.64 (0.24–1.70)	66.9 (55.4–76.7)	0.73 (0.40–1.33)
Routine & manual	96.9 (91.2–98.9)	0.69 (0.14–3.45)	91.4 (85.8–94.9)	0.62 (0.25–1.50)	86.4 (79.6–91.1)	0.51 (0.24–1.12)	65.5 (57.7–72.5)	0.66 (0.41–1.08)
Respondent is main food provider								
No	94.7 (88.0–97.8)	Reference	85.3 (77.3–90.8)	Reference	74.0 (64.8–81.5)	Reference	48.7 (39.4–58.0)	Reference
Yes	98.9 (97.3–99.6)	5.15 (1.40–18.89)	96.9 (94.6–98.3)	5.46 (2.46–12.09)	96.6 (93.4–98.1)	9.86 (4.67–20.83)	78.6 (73.6–82.9)	3.87 (2.42–6.18)

*CI* confidence intervals, *NS*-*SEC* National Statistics socio-economic classification, -- not applicable

Data in Additional file 1: Table S1, S2, S3, S4 and S5 show that more than 75 % of respondents lived in households where the MFP reported confidence with each cooking technique and food, except oily fish (72.7 %, 95 % CI: 68.2–76.7) and pulses (63.0 %; 58.1–67.6). The MFP in more than 90 % of respondent households reported being able to prepare convenience foods (94.7 %, 95 % CI: 91.8–96.5), a complete meal from ready-made ingredients (93.3 %, 95 % CI: 90.4–95.4), and a main dish from basic ingredients (93.2 %, 95 % CI: 90.2–95.4) without help. Very few differences in MFP confidence and ability were seen by respondent socio-demographic characteristics. Where these were seen, those in the youngest age group (19–34 years) and lowest socio-economic group were least likely to live in a household where the MFP reported confidence.

## Discussion

### Summary of results

As far as we are aware, this is the only exploration of the prevalence and socio-demographic correlates of adult cooking skills using recent and population-representative UK data. Our results also contribute to the limited international evidence on this topic. With a few notable exceptions, we found high prevalence of self-reported confidence with using a range of cooking techniques and cooking a range of foods and dishes in both respondents and household MFPs. Almost two-thirds of respondents said they cooked a main meal at least five times per week and more than four-fifths lived in a household where the MFP did so (the MFP was the person with the main responsibility for shopping and preparing food within a household). Almost 90 % of respondents reported being able to cook a main dish from basic ingredients without help, and more than 90 % of respondents lived in a household where the MFP could do so.

Differences in reported cooking confidence across socio-demographic variables were scattered and inconsistent. Where these were found, in general women and respondents who were also MFPs were most likely, and those in the youngest age group (19–34 years) and lowest socio-economic group were least likely, to report confidence. These differences suggest a number of social inequalities in cooking skills according to gender, socio-economic position and age.

### Strengths and limitations of methods

The NDNS aims to recruit a population-representative sample at each wave. We used the study weights provided to reduce biases related to the sampling method and non-response at the respondent level, where this exists. Thus, we are confident that this is the most population-representative data on cooking skills currently available in the UK. Previous quantitative studies have either been restricted to those living in low-income households [9], small convenience samples [30], or were conducted more than 15 years ago [7].

It is possible that self-reported data on cooking skills is subject to social desirability bias [7]. However, it is not clear that this would be stable across socio-demographic groups. For instance, whilst women may feel pressure to report more confidence than they feel with cooking [21], men may not. Thus, our results may over-state gender differences in cooking skills. Although there is evidence of gender differences in the social desirability bias in dietary self-report [36], we are not aware of any studies of social desirability bias in cooking skills specifically. The directions of effect of any other socio-demographic differences in social desirability bias are harder to predict.

The validity and reliability of the questions used to assess cooking skills have not been explored. Differences in individual interpretations of the meaning of ‘confidence’ with cooking techniques and foods are likely to introduce error. Systematic differences in interpretations across socio-economic groups may also exist, leading to bias. Again, it is difficult to predict the directions of any effects.

The complexity of the phenomenon of ‘cooking’ has been noted [19, 20]. It is unlikely that the simple questions used here adequately capture this complex construct. A short questionnaire, with good test-retest reliability and internal consistency has been recently developed [12]. Whilst this shares some similarities with some of the questions used here, it too is simplistic and unlikely to capture the full complexity of cooking skills. Another questionnaire has been developed to assess the impact of cooking skills interventions [37]. However, this focuses on the wider potential outcomes of cooking skills interventions, rather than just the skills themselves–covering issues such as confidence in using a recipe; and frequency of purchasing convenience food and experimenting with new foods. A simple, but comprehensive, measure of cooking skills is required for population monitoring.

Data from wave 1 of the NDNS are now around 5 years old. It is possible, although unlikely, that prevalence of cooking skills has changed substantially over these years. Although more recent waves of the NDNS have been conducted, they did not include questions on cooking skills. Ongoing monitoring of population cooking skills would be valuable.

At just over 500 adults, wave 1 of the NDNS is relatively small. The NDNS is currently conceived as a ‘rolling programme’ with each new wave being combined with previous waves to increase sample size. As indicated above, we were not able to take advantage of more recent waves of data. Nor did we include the approximately 50 % of wave 1 respondents who were children. Although children were asked some questions about cooking in wave 1 of NDNS, these did not cover skills and confidence as in adults. This means data from children could not be combined with that from adults to increase the sample size.

As only 10 % of the NDNS were non-white, we were not able to reliably study ethnic variations in cooking skills. Nor did we study where respondents and MFPs obtained their cooking skills from.

We originally intended to model the relationships between markers of cooking skill and markers of dietary quality and body composition, using multivariate methods. However, as cooking skills were so highly prevalent, and little variability was present, these measures were not effective at differentiating respondents within the population. Future work should explore these relationships further.

### Interpretation and implications of results

Our results suggest very high prevalence of reporting confidence with cooking. Compared to similar data collected in the UK in 1997, reported confidence in cooking all ten food items has increased in both men and women [22]. For example, whilst more than 75 % of respondents were confident with all techniques except stir-frying in the current work, confidence was only this high for boiling, grilling, frying and oven-baking in 1997. Furthermore, gender differences seen in cooking confidence for all ten foods in 1997 were much less evident in the current work [22]. Possible explanations for these improvements include the proliferation of local cooking skills initiatives across the UK [15], increased media coverage of food and cooking topics, and changes in individuals’ perceptions of their own skills.

Our results suggest that most UK adults do not perceive themselves to be lacking cooking skills–or, at least, are not willing to acknowledge this to a researcher. This suggests that most UK adults are unlikely to think (or, perhaps, admit) they would benefit from a basic cooking skills course and so volunteer for such an intervention. Indeed, previous studies have struggled to recruit to evaluations of cooking skills interventions [16]. This is not to say that few individuals would benefit, but any intervention would have to be well-targeted and carefully consider how best to reach and recruit those most likely to benefit.

Despite high prevalence of reported cooking skills, we found that these skills are not necessarily being frequently used. For example, whilst almost 90 % of respondents reported being able to prepare a meal from basic ingredients without help, only two-thirds did so five times a week or more. This confirms that possessing cooking skills is not the same as making frequent use of these skills [7, 16]. One reason why people may not make regular use of their cooking skills is that other members of their household are responsible for cooking. This is reflected in the high frequency of meal preparation amongst MFPs–with over 80 % preparing main meals on five or more days per week.

It seems logical that having cooking skills is a pre-requisite for being able to prepare nutritious meals, and associations between cooking skills and dietary quality have been reported [10–13]. As described above, the high prevalence of reported cooking skills meant we were unable to study any relationships between cooking skills and either dietary quality or adiposity. However, it should not be assumed that possessing cooking skills means that food cooked, or eaten, will necessarily be nutritious. Fresh green vegetables were one of the foods that respondents were most confident with preparing (92.7 % reporting confidence), yet less than 40 % of respondents in wave 1 of NDNS consumed five or more 80 g portions of fruit and vegetables per day [33]. Providing individuals with the skills to cook nutritious meals may be necessary, but not sufficient, to ensure that they do so regularly [38, 39]. In order to have a substantial positive impact on the population’s dietary intake, interventions should also take account of the many social and environmental determinants of diet [19, 40].

Whilst we found few socio-demographic differences in self-reported cooking skills, those that were present tended to indicate that women and respondents who were MFPs were most likely to be confident with cooking, whilst those aged 19–34 years and those in the lowest socio-economic group were least likely to be confident. These findings suggest that any attempt to recruit adults to cooking skills interventions may find it useful to focus on recruiting men, those younger than 35 years, and those in the least affluent socio-economic groups.

Many others have confirmed that women continue to spend more time cooking than men [8, 23–28], so the finding that they report more developed cooking skills is not surprising. As the MFP is the person with the main responsibility for shopping and preparing food in a households, it makes some intuitive sense that this person might also have the most skill and confidence with food preparation.

Age-related differences in cooking skill may reflect differences in frequency of cooking–with the youngest adults being least likely to have, or have had, children at home and so be preparing food frequently and therefore developing their skills. The results give some credence to the popular belief that younger generations have ‘forgotten’ how to cook due to increased exposure to convenience foods [41]. However, it also possible that young adults only develop cooking skills when they perceive these to be required–for example, when they become responsible for their own children. Previous work with small sample sizes has found that older and younger UK women report similar levels of cooking skills–although use these differently [19, 30]. Further work is required to explore how and when individuals develop cooking skills and if there are any particular ‘windows of opportunity’ for intervention.

Where differences were present by socio-economic position, those in the lowest socio-economic group had the lowest cooking confidence. Qualitative research has described a number of barriers to developing cooking skills through experimentation in more deprived groups, including: lack of time, fear of waste, difficulties with following written recipes (perhaps compounded by limited literacy and numeracy), and uncertainties over food safety and labelling [19, 42]. The proportion of adults living in a household where the MFP could cook a meal from basic ingredients (93 % overall) was higher in the current general population sample than in a UK low-income sample (90 % of women and 80 % of men) [9]. This suggests socio-economic inequalities in household cooking skills exist in the UK.

## Conclusions

In this population-representative sample of UK adults, self-reported confidence with using most cooking techniques and preparing most foods was high in respondents and those in their households responsible for food preparation. The great majority of respondents said they were able to prepare a main meal from basic ingredients without help. There were few socio-demographic differences in reported cooking skills, but where these did occur women and those who had primary household responsibility for food tended to be most likely, and those in the lowest age (19–34 years) and socio-economic group least likely, to report confidence. Adult cooking skills interventions should be clearly targeted at those most at risk of reporting low levels of cooking confidence and poorer cooking skills. Careful consideration of how best to reach and recruit those most likely to benefit is required.

## Additional file

Additional file 1: Table S1. Confidence of main food provider in using eight cooking techniques, National Diet & Nutrition Survey, 2008–09, *n* = 490. **Table S2.** Odds of main food provider having confidence in using eight cooking techniques, National Diet & Nutrition Survey, 2008–09, *n* = 490. **Table S3.** Confidence of main food in cooking 10 foods, National Diet & Nutrition Survey, 2008–09, *n* = 490. **Table S4.** Odds of main food provider having confidence in cooking 10 foods, National Diet & Nutrition Survey, 2008–09, *n* = 490. **Table S5.** Ability of main food provider to prepare four dish types without help, National Diet & Nutrition Survey, 2008–09, *n* = 490. (DOC 110 kb)
