# Supplementary material for: Prevalence and socio-demographic correlates of cooking skills in UK adults: cross-sectional analysis of data from the UK National Diet and Nutrition Survey
Source: Int J Behav Nutr Phys Act. 2015 Aug 5;12:99. doi: 10.1186/s12966-015-0261-x (PMC4524366; doi:10.1186/s12966-015-0261-x)
Supplement: Additional file 1: Table S1. — Confidence of main food provider in using eight cooking techniques, National Diet & Nutrition Survey, 2008–09, n = 490. Table S2. Odds of main food provider having confidence in using eight cooking techniques, National Diet & Nutrition Survey, 2008–09, n = 490. Table S3. Confidence of main food in cooking 10 foods, National Diet & Nutrition Survey, 2008–09, n = 490. Table S4. Odds of main food provider having confidence in cooking 10 foods, National Diet & Nutrition Survey, 2008–09, n = 490. Table S5. Ability of main food provider to prepare four dish types without help, National Diet & Nutrition Survey, 2008–09, n = 490. (DOC 110 kb) [file 12966_2015_261_MOESM1_ESM.doc]

Table S1: confidence of main food provider in using eight cooking techniques, National Diet & Nutrition Survey, 2008-09, n=490

| Variable & level | Boiling, % (95% CI) | Steaming, poaching, % (95% CI) | Frying, % (95% CI) | Stir frying, % (95% CI) | Grilling, % (95% CI) | Oven-baking, roasting, % (95% CI) | Stewing, braising, casseroling, % (95% CI) | Microwaving, % (95% CI) |
| --- | --- | --- | --- | --- | --- | --- | --- | --- |
| All respondents | 92.8 (89.7 - 95.0) | 78.1 (73.8 - 81.9) | 85.0 (81.3 - 88.1) | 76.9 (72.6 - 80.7) | 89.3 (85.9 - 92.0) | 92.1 (89.0 - 94.4) | 84.1 (80.3 - 87.3) | 79.5 (75.3 - 83.1) |
| Gender |  |  |  |  |  |  |  |  |
| Men | 90.9 (86.0 - 94.2) | 74.5 (67.7 - 80.3) | 84.9 (79.1 - 89.2) | 77.0 (70.5 - 82.5) | 87.9 (82.8 - 91.7) | 89.2 (84.0 - 92.8) | 83.0 (77.1 - 87.6) | 78.0 (71.6 - 83.2) |
| Women | 94.6 (90.0 - 97.1) | 81.5 (75.8 - 86.1) | 85.1 (79.9 - 89.2) | 76.8 (70.8 - 81.8) | 90.7 (85.7 - 94.0) | 95.0 (90.6 - 97.3) | 85.2 (79.8 - 89.4) | 81.0 (75.2 - 85.6) |
| Age (years) |  |  |  |  |  |  |  |  |
| 19-34 | 89.1 (81.2 - 93.9) | 69.3 (59.8 - 77.4) | 82.9 (74.6 - 88.9) | 73.4 (64.0 - 81.2) | 83.2 (74.7 - 89.3) | 88.8 (80.6 - 93.7) | 75.0 (65.8 - 82.3) | 78.8 (69.2 - 85.9) |
| 35-49 | 94.4 (88.0 - 97.5) | 84.8 (76.8 - 90.4) | 84.2 (76.4 - 89.8) | 82.2 (74.3 - 88.0) | 92.3 (85.9 - 96.0) | 94.8 (89.2 - 97.6) | 86.1 (78.6 - 91.2) | 80.2 (72.7 - 86.0) |
| 50-64 | 95.5 (90.3 - 98.0) | 81.4 (72.5 - 88.0) | 86.6 (79.8 - 91.3) | 80.9 (73.0 - 86.9) | 92.1 (86.4 - 95.6) | 95.0 (89.8 - 97.6) | 90.4 (84.4 - 94.3) | 83.2 (75.6 - 88.8) |
| >64 | 92.5 (82.9 - 96.9) | 77.1 (66.8 - 85.0) | 87.1 (77.2 - 93.0) | 69.6 (58.9 - 78.6) | 90.3 (81.3 - 95.3) | 89.7 (80.4 - 94.9) | 86.5 (76.6 - 92.6) | 75.0 (64.6 - 83.2) |
| NS-SEC |  |  |  |  |  |  |  |  |
| Managerial & prof. | 92.3 (86.8 - 95.7) | 81.9 (74.8 - 87.3) | 86.3 (80.2 - 90.7) | 82.8 (76.4 - 87.7) | 89.4 (83.6 - 93.3) | 92.4 (86.8 - 95.7) | 85.5 (79.4 - 90.0) | 83.2 (76.9 - 88.0) |
| Intermediate | 95.0 (85.4 - 98.4) | 85.7 (76.5 - 91.7) | 90.5 (81.8 - 95.2) | 84.8 (75.5 - 91.0) | 93.7 (85.2 - 97.4) | 96.8 (87.3 - 99.3) | 95.2 (86.9 - 98.3) | 81.6 (71.9 - 88.5) |
| Routine & manual | 92.1 (86.4 - 95.5) | 69.1 (61.3 - 76.0) | 79.8 (72.6 - 85.4) | 65.4 (57.3 - 72.7) | 87.0 (80.7 - 91.5) | 89.6 (83.9 - 93.4) | 77.0 (69.5 - 83.0) | 74.7 (67.1 - 81.0) |

CI: confidence intervals; NS-SEC: National Statistics socio-economic classification

Table S2: odds of main food provider having confidence in using eight cooking techniques, National Diet & Nutrition Survey, 2008-09, n=490

| Variable & level | Boiling, Odds ratio (95% CI) | Steaming, poaching, Odds ratio (95% CI) | Frying, Odds ratio (95% CI) | Stir frying, Odds ratio (95% CI) | Grilling, Odds ratio (95% CI) | Oven-baking, roasting, Odds ratio (95% CI) | Stewing, braising, casseroling, Odds ratio (95% CI) | Microwaving, Odds ratio (95% CI) |
| --- | --- | --- | --- | --- | --- | --- | --- | --- |
| Gender |  |  |  |  |  |  |  |  |
| Men | Reference | Reference | Reference | Reference | Reference | Reference | Reference | Reference |
| Women | 1.74 (0.76 – 3.98) | 1.51 (0.94 – 2.43) | 1.02 (0.60 – 1.74) | 0.99 (0.62 – 1.56) | 1.33 (0.70 – 2.52) | 2.28 (1.02 – 5.09) | 1.18 (0.70 – 2.00) | 1.20 (0.75 – 1.94) |
| Age (years) |  |  |  |  |  |  |  |  |
| 19-34 | Reference | Reference | Reference | Reference | Reference | Reference | Reference | Reference |
| 35-49 | 2.07 (0.73 – 5.88) | 2.48 (1.27 – 4.84) | 1.10 (0.54 – 2.23) | 1.67 (0.88 – 3.16) | 2.43 (1.03 – 5.71) | 2.31 (0.83 – 6.39) | 2.07 (1.04 – 4.10) | 1.09 (0.57 – 2.09) |
| 50-64 | 2.61 (0.92 – 7.34) | 1.94 (1.00 – 3.76) | 1.33 (0.66 – 2.69) | 1.53 (0.81 – 2.89) | 2.37 (1.06 – 5.28) | 2.40 (0.89 – 6.48) | 3.16 (1.55 – 6.42) | 1.34 (0.67 – 2.66) |
| >64 | 1.52 (0.49 – 4.70) | 1.50 (0.77 – 2.91) | 1.39 (0.59 – 3.25) | 0.83 (0.43 – 1.58) | 1.88 (0.75 – 4.76) | 1.11 (0.41 – 2.99) | 2.14 (0.96 – 4.77) | 0.81 (0.40 – 1.64) |
| NS-SEC |  |  |  |  |  |  |  |  |
| Managerial & prof. | Reference | Reference | Reference | Reference | Reference | Reference | Reference | Reference |
| Intermediate | 1.58 (0.42 – 5.96) | 1.33 (0.63 – 2.80) | 1.51 (0.64 – 3.58) | 1.16 (0.57 – 2.37) | 1.75 (0.60 – 5.09) | 2.50 (0.50 – 12.49) | 3.34 (1.04 – 10.72) | 0.90 (0.45 – 1.78) |
| Routine & manual | 0.96 (0.41 – 2.26) | 0.50 (0.29 – 0.89) | 0.63 (0.35 – 1.13) | 0.39 (0.23 – 0.66) | 0.79 (0.40 – 1.57) | 0.70 (0.32 – 1.55) | 0.57 (0.32 – 1.00) | 0.60 (0.35 – 1.03) |

CI: confidence intervals; NS-SEC: National Statistics socio-economic classification

Table S3: confidence of main food in cooking 10 foods, National Diet & Nutrition Survey, 2008-09, n=490

| Variable & level | Red meat, % (95% CI) | Chicken, % (95% CI) | White fish, % (95% CI) | Oily fish, % (95% CI) | Pulses, % (95% CI) | Dry pasta, % (95% CI) | Rice (savoury), % (95% CI) | Potatoes (not chips) , % (95% CI) | Fresh green veg, % (95% CI) | Root veg, % (95% CI) |
| --- | --- | --- | --- | --- | --- | --- | --- | --- | --- | --- |
| All | 87.3 (83.7 - 90.2) | 90.8 (87.4 - 93.3) | 82.1 (77.9 - 85.6) | 72.7 (68.2 - 76.7) | 63.0 (58.1 - 67.6) | 84.5 (80.5 - 87.8) | 86.8 (83.0 - 89.8) | 92.7 (89.4 - 95.1) | 93.0 (89.7 - 95.3) | 90.5 (87.1 - 93.1) |
| Gender |  |  |  |  |  |  |  |  |  |  |
| Men | 88.0 (82.6 - 91.8) | 89.6 (84.5 - 93.2) | 80.2 (73.6 - 85.5) | 69.3 (62.2 - 75.5) | 60.5 (53.1 - 67.4) | 80.0 (73.6 - 85.2) | 83.5 (77.4 - 88.2) | 91.0 (85.7 - 94.5) | 89.8 (84.3 - 93.5) | 88.6 (83.2 - 92.5) |
| Women | 86.7 (91.4 - 90.6) | 91.9 (87.0 - 95.0) | 83.9 (78.5 - 88.1) | 75.9 (70.1 - 80.9) | 65.4 (58.8 - 71.4) | 88.8 (83.7 - 92.5) | 89.9 (84.9 - 93.4) | 94.4 (89.5 - 97.1) | 96.1 (91.4 - 98.3) | 92.3 (87.5 - 95.4) |
| Age (years) |  |  |  |  |  |  |  |  |  |  |
| 19-34 | 79.2 (70.5 - 85.9) | 85.5 (77.4 - 91.0) | 73.1 (63.7 - 80.9) | 60.6 (50.7 - 69.7) | 51.0 (41.0 - 60.9) | 85.8 (77.2 - 91.6) | 87.4 (79.4 - 92.6) | 90.5 (82.0 - 95.2) | 91.5 (83.4 - 95.8) | 84.2 (75.5 - 90.2) |
| 35-49 | 88.7 (81.1 - 93.5) | 90.4 (83.0 - 94.8) | 77.7 (68.9 - 84.5) | 74.5 (65.9 - 81.6) | 66.1 (57.2 - 73.9) | 88.8 (80.8 - 93.8) | 89.3 (81.2 - 94.2) | 92.5 (84.5 - 96.5) | 92.5 (84.5 - 96.5) | 92.0 (84.8 - 96.0) |
| 50-64 | 94.2 (88.9 - 97.0) | 95.1 (89.7 - 97.7) | 91.8 (85.8 - 95.4) | 82.2 (74.7 - 87.9) | 66.9 (57.3 - 75.2) | 85.8 (78.0 - 91.1) | 88.0 (80.5 - 92.8) | 95.9 (89.6 - 97.6) | 94.5 (89.1 - 97.3) | 94.7 (89.8 - 97.4) |
| >64 | 88.2 (79.0 - 93.7) | 93.3 (84.0 - 97.4) | 88.7 (78.6 - 94.4) | 75.3 (64.6 - 83.6) | 70.6 (59.8 - 79.6) | 75.3 (65.0 - 83.3) | 80.9 (70.7 - 88.1) | 93.5 (84.4 - 97.5) | 93.9 (84.5 - 97.7) | 92.1 (82.6 - 96.6) |
| NS-SEC |  |  |  |  |  |  |  |  |  |  |
| Managerial | 86.5 (80.1 - 91.0) | 88.3 (82.0 - 92.6) | 83.1 (76.4 - 88.2) | 77.0 (70.1 - 82.8) | 71.2 (63.6 - 77.8) | 85.6 (79.1 - 90.4) | 88.5 (82.1 - 92.9) | 90.9 (85.0 - 94.6) | 92.0 (86.1 - 95.5) | 90.9 (85.0 - 94.7) |
| Intermediate | 92.8 (84.6 - 96.8) | 96.8 (88.1 - 99.2) | 90.7 (82.5 - 95.3) | 80.5 (70.9 - 87.5) | 68.3 (57.0 - 77.8) | 90.9 (82.0 - 95.6) | 94.1 (86.0 - 97.6) | 95.4 (83.5 - 98.8) | 97.4 (87.6 - 99.5) | 96.6 (87.0 - 99.2) |
| Routine | 85.3 (78.8 - 90.1) | 89.9 (83.9 - 93.8) | 76.4 (68.5 - 82.8) | 64.0 (55.9 - 71.4) | 50.7 (42.6 - 58.8) | 77.9 (70.3 - 84.1) | 79.7 (72.3 - 85.5) | 92.7 (86.7 - 96.2) | 92.1 (86.0 - 95.6) | 86.9 (80.5 - 91.5) |

CI: confidence intervals; NS-SEC: National Statistics socio-economic classification

Table S4: odds of main food provider having confidence in cooking 10 foods, National Diet & Nutrition Survey, 2008-09, n=490

| Variable & level | Red meat, Odds ratio (95% CI) | Chicken, Odds ratio (95% CI) | White fish, Odds ratio (95% CI) | Oily fish, Odds ratio (95% CI) | Pulses, Odds ratio (95% CI) | Dry pasta, Odds ratio (95% CI) | Rice (savoury), Odds ratio (95% CI) | Potatoes (not chips) , Odds ratio (95% CI) | Fresh green veg, Odds ratio (95% CI) | Root veg, (95% CI) |
| --- | --- | --- | --- | --- | --- | --- | --- | --- | --- | --- |
| Gender |  |  |  |  |  |  |  |  |  |  |
| Men | Reference | Reference | Reference | Reference | Reference | Reference | Reference | Reference | Reference | Reference |
| Women | 0.89 (0.49 – 1.60) | 1.30 (0.65 – 2.62) | 1.28 (0.77 – 2.15) | 1.40 (0.91 – 2.16) | 1.23 (0.82 – 1.86) | 1.98 (1.12 – 3.50) | 1.76 (0.96 – 3.22) | 1.65 (0.70 – 3.93) | 2.80 (1.07 – 7.33) | 1.55 (0.76 – 3.15) |
| Age (years) |  |  |  |  |  |  |  |  |  |  |
| 19-34 | Reference | Reference | Reference | Reference | Reference | Reference | Reference | Reference | Reference | Reference |
| 35-49 | 2.06 (0.96 – 4.42) | 1.60 (0.68 – 3.77) | 1.28 (0.68 – 2.39) | 1.90 (1.07 – 3.39) | 1.87 (1.08 – 3.25) | 1.31 (0.55 – 3.13) | 1.21 (0.50 – 2.93) | 1.29 (0.43 – 3.89) | 1.15 (0.38 – 3.50) | 2.17 (0.87 – 5.41) |
| 50-64 | 4.24 (1.82 – 9.88) | 3.28 (1.25 – 8.57) | 4.09 (1.92 – 8.68) | 3.01 (1.65 – 5.50) | 1.94 (1.09 – 3.45) | 0.99 (0.45 – 2.19) | 1.05 (0.46 – 2.39) | 1.97 (0.67 – 5.74) | 1.59 (0.55 – 4.60) | 3.39 (1.38 – 8.33) |
| >64 | 1.96 (0.85 – 4.52) | 2.37 (0.77 – 7.27) | 2.87 (1.20 – 6.90) | 1.99 (1.03 – 3.82) | 2.31 (1.12 – 4.34) | 0.50 (0.23 – 1.08) | 0.61 (0.27 – 1.37) | 1.52 (0.44 – 5.21) | 1.42 (0.40 – 5.03) | 2.18 (0.77 – 6.18) |
| NS-SEC |  |  |  |  |  |  |  |  |  |  |
| Managerial | Reference | Reference | Reference | Reference | Reference | Reference | Reference | Reference | Reference | Reference |
| Intermediate | 2.03 (0.77 – 5.38) | 4.00 (0.90 – 17.86) | 1.99 (0.86 – 4.60) | 1.23 (0.65 – 2.34) | 0.87 (0.48 – 1.58) | 1.67 (0.68 – 4.12) | 2.06 (0.70 – 6.10) | 2.07 (0.45 – 9.51) | 3.30 (0.55 – 19.86) | 2.84 (0.60 – 13.55) |
| Routine | 0.91 (0.48 – 1.73) | 1.17 (0.57 – 2.44) | 0.66 (0.37 – 1.17) | 0.53 (0.32 – 0.87) | 0.42 (0.26 – 0.67) | 0.59 (0.32 – 1.09) | 0.51 (0.26 – 0.99) | 1.28 (0.53 – 3.09) | 1.01 (0.41 – 2.45) | 0.66 (0.31 – 1.40) |

CI: confidence intervals; NS-SEC: National Statistics socio-economic classification

Table S5: ability of main food provider to prepare four dish types without help, National Diet & Nutrition Survey, 2008-09, n=490

| Variable & level | Convenience foods & ready meals | | Complete meal from ready-made ingredients | | Main dish from basic ingredients | | Cake or biscuits from basic ingredients | |
| --- | --- | --- | --- | --- | --- | --- | --- | --- |
|  | % (95% CI) | Odds ratio (95% CI) | % (95% CI) | Odds ratio (95% CI) | % (95% CI) | Odds ratio (95% CI) | % (95% CI) | Odds ratio (95% CI) |
| All respondents | 94.7 (91.8 - 96.5) | -- | 93.3 (90.4 - 95.4) | -- | 93.2 (90.2 - 95.4) | -- | 79.1 (94.9 - 82.8) | -- |
| Gender |  |  |  |  |  |  |  |  |
| Men | 92.7 (88.1 - 95.5) | Reference | 91.8 (87.2 - 94.9) | Reference | 92.0 (87.1 - 95.1) | Reference | 70.3 (63.4 - 76.4) | Reference |
| Women | 96.6 (92.2 - 98.5) | 2.25 (0.81 – 6.24) | 94.7 (90.4 - 97.2) | 1.60 (0.70 – 3.62) | 94.4 (90.1 - 96.9) | 1.48 (0.65 – 3.34) | 87.6 (82.6 - 91.4) | 2.99 (1.80 – 4.97) |
| Age (years) |  |  |  |  |  |  |  |  |
| 19-34 | 92.2 (84.7 - 96.2) | Reference | 91.9 (84.4 - 95.9) | Reference | 90.4 (82.8 - 94.8) | Reference | 75.0 (65.5 - 82.6) | Reference |
| 35-49 | 96.4 (90.8 - 98.7) | 2.28 (0.65 – 8.04) | 95.4 (89.9 - 98.0) | 1.85 (0.60 – 5.70) | 93.0 (86.1 - 96.6) | 1.42 (0.51 – 3.91) | 80.8 (72.6 - 86.9) | 1.40 (0.73 – 2.68) |
| 50-64 | 96.3 (91.6 - 98.4) | 2.22 (0.70 – 7.01) | 94.5 (89.1 - 97.4) | 1.54 (0.54 – 4.39) | 97.0 (92.4 - 98.8) | 3.38 (1.05 – 10.86) | 82.5 (74.5 - 88.3) | 1.57 (0.81 – 3.04) |
| >64 | 93.7 (84.5 - 97.6) | 1.27 (0.36 – 4.47) | 90.9 (81.8 - 95.7) | 0.89 (0.30 – 2.64) | 92.9 (84.2 - 97.0) | 1.40 (0.46 – 4.27) | 78.7 (68.6 - 86.2) | 1.23 (0.61 – 2.47) |
| NS-SEC |  |  |  |  |  |  |  |  |
| Managerial & prof. | 91.9 (86.3 - 95.4) | Reference | 90.3 (84.5 - 94.1) | Reference | 92.0 (86.4 - 95.4) | Reference | 82.1 (75.5 - 87.1) | Reference |
| Intermediate | 97.1 (87.7 - 99.4) | 2.96 (0.56 – 15.66) | 97.9 (86.4 - 99.7) | 4.93 (0.63 – 38.31) | 94.4 (84.6 - 98.2) | 1.47 (0.41 – 5.25) | 78.0 (67.0 - 86.0) | 0.77 (0.39 – 1.53) |
| Routine & manual | 96.3 (91.7 - 98.4) | 2.27 (0.81 – 6.40) | 93.8 (89.0 - 96.6) | 1.62 (0.71 – 3.68) | 94.4 (89.7 - 97.0) | 1.45 (0.60 – 3.51) | 78.3 (71.2 - 84.1) | 0.79 (0.46 – 1.37) |

CI: confidence intervals; NS-SEC: National Statistics socio-economic classification
